# Evaluation of the frequency and risk factors of spontaneous intramuscular hemorrhage associated with dermatomyositis

**DOI:** 10.1007/s00296-024-05612-6

**Published:** 2024-05-29

**Authors:** Döndü Üsküdar Cansu, Reşit Yildirim, Burcu Ceren Uludoğan, Muzaffer Bilgin, Cengiz Korkmaz

**Affiliations:** 1 Division of Rheumatology, Department of Internal Medicine, Eskişehir Osmangazi University, Eskişehir, Turkey; 2 Department of Biostatistics, Eskişehir Osmangazi University, Eskişehir, Turkey; 3 Department of Rheumatology, School of Medicine, Eskişehir Osmangazi University Eskisehir, Eskişehir, 26480 Turkey

**Keywords:** Dermatomyositis, Spontaneous intramuscular hemorrhage, Intramuscular hemorrhage, Muscle hematoma

## Abstract

Dermatomyositis (DM) is an idiopathic inflammatory myositis (IIM) characterized by skin manifestations and muscle involvement. Spontaneous intramuscular hemorrhage (SIH) is a fatal complication that is very rare in the course of DM, but not well known to rheumatologists. Our aim was to determine the frequency and possible risk factors of DM-related SIH. A retrospective analysis was conducted on a cohort of DM patients who were observed in the rheumatology department of the university hospital between 1998 and January 2024. The clinical, laboratory, radiological data of the patients and the treatments they received during the follow-up were analyzed. To determine possible risk factors for the development of SIH in the course of DM, our patients with DM were analyzed together with other rare SIH cases in the literature. The study included 42 of our DM patients. 32 of the patients (76.2%) were female. The median age of the patients was 53 (24–82) years, the median age of DM diagnosis of the patients was 47 (18–75) years, and the median duration of DM of the patients was 36 (2-276) months. 7.1% of patients had dysphagia, and 16.7% had intertitial lung disease (ILD). 5 (11.9%) patients were diagnosed with malignancy. The incidence rate of SIH development in our DM cohort was 0.238/100 patient years (95% CI 0.006–1.256). We tried to identify independent risk factors for SIH development by comparing our 41 DM patients without SIH with the data of patients with 23 DM-related SIH collected from the literature by adding our 1 patient (24 pts). Male sex (OR 4.97, 95% CI 1.66–14.92, *p* = 0.003), ILD presence (OR 9.71, 95% CI 2.99–31.47, *p* < 0.001), anti-MDA5 positivity (OR 16.0, 95% CI 1.60-159.3, *p* = 0.006), anti-Ro52 positivity (OR 11.6, 95% CI 2.93–46.34, *p* < 0.001), heparin use (OR 4.42, 95% CI 2.68–7.24, *p* < 0.001), intravenous immunoglobulin (IVIG) use (OR 11.7, 95% CI 2.26–60.54, *p* < 0.001), and steroid dose (OR 1.03, 95% CI 1.00-1.05, *p* = 0.005) were identified as risk factors for the development of SIH in the univariate analysis. The death rate due to hemorrhage was 50%. No single risk factor was found to be associated with death. As a result, SIH may occasionally arise in patients with DM. Rheumatologists should be aware that patients with dysphagia and/or ILD, who are on heparin, getting high doses of steroids, and test positive for anti-MDA5 and/or anti-Ro52 antibodies may develop SIH in the early stages of DM.

## Introduction

Idiopathic inflammatory myopathies (IIM) are a heterogeneous group of diseases characterized by muscle weakness as a result of chronic inflammation of the skeletal muscle. Other organs such as the skin, joints, lungs and heart are also affected in IIMs, contributing to morbidity and mortality. IIM patients with typical skin rash, such as heliotrope rash, Gottron papule, are classified as dermatomyositis (DM) [1]. A significant improvement for IIMs has been the identification of myositis-specific autoantibodies (MSAs) that help in the diagnosis and classification of IIMs. Anti-melanoma differentiation-associated gene 5 (MDA5) antibodies are often associated with ulcerations in interphalangeal joints in DM patients, as well as an increase in the incidence of intertitial lung disease (ILD) [1, 2]. Other than MSAs, myositis-related antibodies such as anti-Ro52, anti-PM-Scl, anti-Ku, and anti-U1RNP have also been identified [3].

Pneumomediastinum is a rare but still known complication in the course of DM, while DM-related spontaneous intramuscular bleeding (SIH) is very rarely defined and is not very well known among rheumatologists. So far only case reports have been identified in the literature [4–18]. The frequency and risk factors of DM-related SIH are unknown. There is not enough information about the diagnostic method and treatment. A recent publication also suggested that this complication, which could be fatal, may be associated with MDA5 antibody [4].

In the literature, only a case-based literature review examined prognostic factors for death in patients with DM-related SIH, while possible risk factors for the development of SIH were not investigated [5]. We aimed to alert rheumatologists and clinicians interested in IIM about SIH and ascertain the frequency of SIH as well as potential risk factors.

## Methods

### Study population

This retrospective study consisted of a DM cohort followed in a university hospital, department of rheumatology, from 1998–January 2024, meeting the 1975 Bohan Peter classification criteria for DM [19]. Amiopathic DM, non-DM IIMs, and patients under 18 years of age were not included in the study. For the study, approval of ethics was obtained from the Eskisehir Osmangazi University Ethics Committee.

### Patient characteristics

The clinical, laboratory, and radiological data of patients were evaluated. DM diagnosis age, DM follow-up time, gender, presence of dysphagia during the diagnosis of DM, skin, muscle, lung and heart involvement, presence of malignancy, laboratory parameters at the time of diagnosis [creatinine level, total protein, albumin, creatine kinase (CK), lactate dehydrogenase (LDH), aspartate aminotransferase (AST), alanine aminotransferase (ALT) levels, complete blood count, C-reactive protein (CRP), erythrocyte sedimentation rate (ESR), anti-nuclear antibody (ANA), myositis specific antibody (MSA) results, anti-Ro52 results, prothrombin time (PT), activated partial thromboplastin time (aPTT), fibrinogen levels], steroid dose used during the disease course, immunosuppressive drugs, intravenous immunoglobulin (IVIG) use, antiagregan and heparin use were recorded.

The imaging techniques utilized to diagnose hematomas, the duration of hematoma development, and the localization of hematomas (upper or lower extremity, retroperitoneum, iliopsoas, and deep muscular hematomas) were evaluated in patients who developed hematomas. The administration of heparin or antiagregan, dosage and use of steroids, IVIG administration, hematoma treatments, and hematoma outcomes were all documented. We evaluated additional cases of DM-related SIH identified in the literature in addition to our own in order to identify risk factors.

### Statistical analysis

Continuous data are given as median (min-max), while categorical data are given as percentage (%). The Shapiro–Wilk test was used to compare the conformity of the data to normal distribution. For the comparison of groups that do not have a normal distribution, Mann–Whitney U test was used. In the analysis of cross tables, Pearson Chi-square, Pearson exact Chi-square, Fisher’s exact Chi-square analyses were used. A logistic regression model was used to determine the clinical, laboratory and treatment variables which are independently associated with the incidence of SIH, presenting the adjusted odds ratio (OR) and the confidence interval (CI) of 95%. The incidence rate of SIH was calculated as per 100 patient years. *p* < 0.05 was regarded as statistically significant. Analyses were performed using IBM SPSS Statistics 21.0 (IBM Corp. Released 2012. IBM SPSS Statistics for Windows, Version 21.0. Armonk, NY: IBM Corp.) program.

## Results

### Demographic findings of our dermatomyositis cohort

The study included 42 of our DM patients. 32 of the patients (76.2%) were female. The median DM diagnosis age of the patients was 47 (18–75) years, and the median duration of DM was 36 (2-276) months. 7.1% of patients had dysphagia, 16.7% had ILD and 2.4% had cardiac involvement. 5 (11.9%) patients had malignancy. Clinical features of our patients are given in Table 1.

**Table 1:** Clinical characteristics of our dermatomyositis cohort at the time of diagnosis

	All DM group (n = 42)
Diagnostic age of DM, median, (min–max), years	47 (18–75)
Age, median, (min–max), years	53 (24–82)
Gender, Female/Male	32/10
DM disease duration, median, (min–max), months	36 (2-276)
Comorbidity, % (n)	21.4% (9)
Dysphagia, % (n)	7.1% (3)
ILD, % (n)	16.7% (7)
Cardiac involvement, % (n)	2.4% (1)
Malignancy, % (n)	11.9% (5)
Malignancy type, n	Lung cancer *n* = 2 Ovarian cancer *n* = 1 Colorectal cancer *n* = 1 Nasopharyngeal cancer *n* = 1
*Treatment at diagnosis*	
Acetylsalicylic acid, % (n)	11.9% (5)
Heparin, % (n)	2.4% (1)
Steroid, % (n)	100% (42)
Steroid dose, median, (min–max), mg/day	48 (25-1000)
AZA, % (n)	57.1% (24)
MMF, % (n)	4.8% (2)
MTX, % (n)	23.8% (10)
IVIG, % (n)	4.8% (2)
Spontaneous intramuscular hemorrhage, % (n)	2.4% (1)
Hematoma localization, % (n)	Bilateral lower extremity
Death at DM diagnosis, % (n)	-
Hematoma-related death, % (n)	-

AZA; azathioprine, DM; dermatomyositis, ILD; interstitial lung disease, IVIG; intravenous immunoglobulin, MMF; mycophenolate mofetil, MTX; methotrexate

### Laboratory findings of our dermatomyositis cohort at the time of diagnosis

At the time of DM diagnosis, laboratory parameters were as follows: median CK level 454 (37-16729) IU/L, AST 55 (21–535) IU/L, CRP 5.9 (0.5–120) mg/dl, and hemoglobin (Hb) 13.3 (8.5–16.2) gr/dl. In our DM cohort, AST level was high in 54.7% (*n* = 23) of our patients, ALT level was high in 54.7% (*n* = 23), CK level was high in 73.8% (*n* = 31), LDH was high in 76.1% (*n* = 32), CRP level was high in 45.2% (*n* = 19), and ESR was high in 42.8% (*n* = 18) of our patients. During the diagnosis of 28 patients, there was no PT or aPTT prolongation. The fibrinogen levels were normal in all patients. In 52.5% of patients, ANA was positive. MSAs positivity was 26.2%. Myositis-specific or associated antibody results are given in Table 2.

**Table 2:** Laboratory results of our dermatomyositis cohort at the time of diagnosis

N	42
Creatinine, median, (min–max), mg/dl	0.58 (0.24–1.8)
Total protein, median, (min–max), mg/dl	7.1 (5.6–8.9)
Albumin, median, (min–max), mg/dl	4 (2.8–4.87)
AST, median, (min–max), IU/L	55 (21–535)
ALT, median, (min–max), IU/L	46 (15–279)
CK, median, (min–max), IU/L	454 (37-16729)
LDH, median, (min–max), IU/L	547 (114–1840)
CRP, median, (min–max), mg/dl	5.9 (0.5–120)
ESR, median, (min–max), mm/hour	23 (6-104)
Hb, median, (min–max), gr/dl	13.3 (8.5–16.2)
WBC, median, (min–max), /mm^3^	7900 (3930–19,400)
PLT, median, (min–max), /mm^3^	125,000 (140,000–519,000)
Prolonged PT	0/28
Prolonged aPTT	0/28
Low fibrinogen level	0/26
ANA positivity, % (n)	*52.5% (21/40)
Myositis specific autoantibody positivity, % (n)	26.2% (11)
*Antibody results*	
Anti-Jo1 positivity, % (n)	2.4% (1/42)
Anti-Mi2 positivity, % (n)	*6.6% (1/15)
Anti-MDA5 positivity, % (n)	*6.6% (1/15)
Anti-TIF1 positivity, % (n)	*20% (3/15)
Anti-SAE1 positivity, % (n)	*13.3% (2/15)
Anti-PL-12 positivity, % (n)	*13.3% (2/15)
Anti-Ro52 positivity, % (n)	11.9% (5/42)

ALT; alanine aminotransferase, ANA; antinuclear antibodies, aPTT; activated partial thromboplastin time, AST; aspartate aminotransferase, CK; creatine phosphokinase, CRP; C-reactive protein, ESR; erythrocyte sedimentation rate, Hb; hemoglobin, LDH; lactate dehydrogenase, PLT; platelet count, PT; prothrombin time, WBC; white blood cell

* Percentages are based on the total number of patients tested

### Treatments given to our dermatomyositis cohort at the time of diagnosis

All patients were given steroids. The median steroid dose was 48 (25-1000) mg/day. When evaluated in terms of immunosuppressive drugs, 57.1% of the patients received azathioprine (AZA) and 23.8% received methotrexate (MTX). 2 (4.8%) patients were given IVIG. 11.9% of patients received acetylsalicylic acid due to comorbid conditions, while 1 (2.4%) received low molecular weight heparin for deep vein thrombosis proflaxis. Medications given during the diagnosis of DM are given in Table 1.

### Spontaneous intramuscular hemorrhage development incidence rate and patient characteristics

Of our 42 DM patients, only 1 (2.4%) had developed SIH. In our study, the incidence rate of SIH in patients with DM was 0.238/100 patient years (95% CI 0.006–1.256).

*Characteristics of the patient developing SIH in our cohort*: 72-year-old male patient was diagnosed with DM due to skin rash (heliotrope rash, Gottron papule), muscle weakness, dysphagia and mass in the lungs for 2 months. Low molecular weight heparin was started for deep vein thrombosis prophylaxis. Methyl prednisolone dosage was in a dose of 40 mg/day intravenously. The seventh day of follow-up, bilateral SIH was detected in both legs by ultrasonography (USG). A 2-gram Hb drop was detected. 2 units of erythrocyte suspension (ES) and supportive therapy were applied. No pathology was detected in abdominal computed tomography (CT), which was taken due to possible intra-abdominal bleeding. Bronchoscopic biopsy confirmed lung adenocarcinoma. The patient’s hematoma regressed in the following days, and no new hematomas developed.

### Risk factors for the development of dermatomyositis-related spontaneous intramuscular hemorrhage

In our DM cohort, we have detected SIH in only 1 patient. We therefore wanted to evaluate the reported SIH cases in the literature to determine the risk factors for SIH in DM patients.

### Literature search

We used the MEDLINE, Web of Science and Scopus databases to identify patients with SIH [20]. We have searched databases up to January 31, 2024, without restrictions for the beginning of the search, using the keywords dermatomyositis [AND] hemorrhage [OR] hematoma [OR] spontaneous intramuscular hemorrhage [OR] intramuscular hematoma [OR] spontaneous bleeding [OR] hemorrhagic myositis. Case series and case presentations were included in the study. Publications containing non-DM inflammatory myositis cases, cases without spontaneous bleeding, poster presentations, publications containing under-18 cases, and articles not written in English were excluded from analysis. Including 23 cases that met the inclusion criteria and our own case, total of 24 SIH patients were analyzed [5–18]. We compared the data of these patients with our 41 DM patients without SIH.

### Characteristics of patients who develop dermatomyositis-associated spontaneous intramuscular hemorrhage

Of the 24 SIH patients, 41.6% were women and their median age was 56.5 (24–80) years. Hematoma appeared a median of 1 (0.3-9) month of DM. 25% of patients had dysphagia, 66.7% had ILD, and 12.5% had malignancy. 76.2% of patients had MSAs positivity. Anti-Jo1 positivity was 5.3%, anti-MDA5 positivity was 57.1%, and anti-Ro52 positivity was 62.5%. The median steroid dose of patients was 120 (40-1000) mg/day, while the IVIG received patient rate was 37.5%. The use of heparin in the whole group was 50% and the use of acetylsalicylic acid was 8.3%. Hematoma developed on the median day of 9.5 (4–30) of heparin treatment. Heparin was stopped in 7 of 12 patients receiving heparin. The frequency of dysphagia (*p* = 0.004) and ILD (p = < 0.001) were greater in DM patients with SIH than those who did not have SIH. SIH was observed more often in men (*p* = 0.007). In patients with SIH, both anti-MDA5 (*p* = 0.013) and anti-Ro52 (*p* < 0.001) positivity were more common. Comparison of patients with DM with and without SIH is given in Table 3.

**Table 3:** Clinical and laboratory characteristics of patients with (together with our patient and cases in the literature) and without (our dermatomyositis cohort) spontaneous intramuscular hemorrhage [5–18]

	DM without SIH (Our cohort)	DM with SIH	*P* values
N	41	24	
Diagnostic age of DM, median, (min–max), years	47 (18–68)	56.5 (24–80)	0.838
Gender, Female/Male	32/9	10/14	0.007
DM disease duration, median, (min–max), months	36 (2-276)	1 (0.3-9)	< 0.001
Dysphagia, % (n)	4.9% (2)	25% (6)	0.004
ILD, % (n)	17.1% (7)	66.7% (16)	< 0.001
Malignancy, % (n)	9.8% (4)	12.5% (3)	0.731
*Laboratory results*			
AST, median, (min–max), IU/L	54 (21–535)	337 (109–1006)	0.064
ALT, median, (min–max), IU/L	47 (15–279)	171 (40–569)	< 0.001
CK, median, (min–max), IU/L	425 (37-16728)	1389 (302-17711)	0.612
LDH, median (min-max), IU/L	551 (114–1840)	711 (404–2268)	0.345
Prolonged aPTT, n	0/27	3/16	0.045
ANA positivity, % (n)	51.3% (20/39)	46.7% (7/15)	0.761
Myositis specific autoantibody	26.8% (11/40)	76.2% (16/21)	< 0.001
Anti-Jo1 positivity, % (n)	2.5% (1/39)	5.3% (1/19)	0.544
Anti-MDA5 positivity, % (n)	7.7% (1/13)	57.1% (8/14)	0.013
Anti-TIF1 positivity, % (n)	25% (3/9)	7.1% (1/14)	0.306
Anti-Ro52 positivity, % (n)	12.5% (5/40)	62.5% (10/16)	< 0.001
*Treatment at DM diagnosis*			
Steroid dose, median (min-max), mg/day	48 (25-1000)	120 (40-1000)	< 0.001
IVIG, % (n)	4.9% (2)	37.5% (9)	< 0.001
Acetylsalicylic acid, % (n)	12.2% (5)	8.3% (2)	0.628
Heparin, % (n)	-	50% (12)	< 0.001

ALT; alanine aminotransferase, ANA; antinuclear antibodies, AST; aspartate aminotransferase, aPTT; activated partial thromboplastin time, CK; creatine phosphokinase, DM; dermatomyositis, Hb; hemoglobin, ILD; interstitial lung disease, IVIG; intravenous immunoglobulin, LDH; lactate dehydrogenase, MSA; myositis specific autoantibody, PLT; platelet count, SIH; spontaneous intramuscular hemorrhage

CT (87.5%) and angiography (29.2%) were used to determine the hematoma. The most common affected region was iliopsoas (41.7%). During the hematoma, the median Hb level was 7.15 (4.1–13.4) gr/dl, the median platelet (PLT) level was 58,500 (50,000–321,000) /mm^3^, and the median CK level was 4210 (151–8270) IU/L. The requirement for ES transfusion was observed in 15 (62.5%) patients with SIH. They were exposed to median 2 (2–20) units of ES transfusion. Embolization was performed in 5 patients, while one patient received a brachial arterial bypass graft. In a group of 24 individuals, the mortality rate due to hematoma or hemorrhage-related events was 50%, and the overall mortality rate, including all causes, was 62.5%. While 12 of the 15 patients died due to bleeding, 2 of the other 3 patients died due to ILD and 1 died due to lung infection. Detailed findings regarding hemorrhage, treatment and outcomes of patients who developed SIH are given in Table 4.

**Table 4:** Detailed hemorrhage-related characteristics of patients with spontaneous intramuscular hemorrhage (together with our patient and cases in the literature) [5–18]

	During intramuscular hemorrhage
n	24
*Imaging for hemorrhage diagnosis*	
USG, % (n)	4.2% (1)
MRI, % (n)	16.7% (4)
CT, % (n)	87.5% (21)
Angiography, % (n)	29.2% (7)
*Hemorrhage localization*	
Upper extremity, % (n)	16.7% (4)
Lower extremity, % (n)	12.5% (3)
Deep muscular hematoma, % (n)	79.2% (19)
Obliqus internus abdominalis, % (n)	4.2% (1)
Retroperitoneal, % (n)	12.5% (3)
Iliopsoas, % (n)	41.7% (10)
*Drugs using during hematoma/ hemorrhage*	
Heparin use, % (n)	50% (12)
Duration of use of heparin, median (min-max), day	9.5 (4–30)
Hb during hematoma, median (min-max), gr/dL	7.15 (4.1–13.4)
PLT during hematoma, median (min-max), /mm^3^	58,500 (50,000–321,000)
CK during hematoma, median (min-max), IU/L	4210 (151–8270)
*Treatments given for hematoma*	
Need for erythrocytes, % (n)	62.5% (15)
Amount of ES administered, median (min-max)	2 (2–20)
Heparin discontinued, % (n)	58.3% (7)
Angiographic embolization, % (n)	20.8% (5)
Hemorrhage -related death, % (n)	50% (12)
All deaths, % (n)	62.5% (15)

CK; creatine phosphokinase, CT; computed tomography, ES; erythrocyte suspension, Hb; hemoglobin, MRI; magnetic resonance imaging, PLT; platelet count, USG; ultrasonography

### Risk factors for the development of dermatomyositis-related spontaneous intramuscular hemorrhage

Risk factors for the development of DM-related SIH according to the univariate analysis are as follows: male gender (OR 4.97, 95% CI 1.66–14.92, *p* = 0.003) and ILD presence (OR 9.71, 95% CI 2.99–31.47, *p* < 0.001); anti-MDA5 positivity (OR 16.0, 95% CI 1.60-159.3, *p* = 0.06); anti-Ro52 positivity (OR 11.6, 95% CI 2.93–46.34, *p* < 0.001); heparin use (OR 4.42, 95% CI 2.68–7.24, *p* < 0.001); and steroid dose (OR 1.03, 95% CI 1.00-1.05, *p* = 0.005). All risk factors are given in Table 5.

**Table 5:** Risk factors for the development of spontaneous intramuscular hemorrhage associated with dermatomyositis (together with our patient and cases in the literature [24 patients in total]) (Univariate analysis) [5–18]

	At the time of DM diagnosis	OR	Lower-upper CI	*p* values
Clinical	Male	4.97	1.66–14.92	0.003
	Age at diagnosis of DM	1.07	1.02–1.12	0.003
	DM disease duration (months)	1.67	1.17–2.38	0.004
	Presence of ILD	9.71	2.99–31.47	< 0.001
	Presence of dysphagia	6.50	1.19–35.40	0.017
Laboratory	ALT level	1.01	1.00-1.02	0.005
	AST level	1.01	1.00-1.02	0.011
	PLT level	1.01	1.00-1.02	0.008
	Presence of MSAs	8.72	2.59–29.52	< 0.001
	Anti-MDA5 positivity	16.0	1.60-159.3	0.006
	Anti-Ro52 positivity	11.6	2.93–46.34	< 0.001
Treatment	Heparin use	4.42	2.68–7.24	< 0.001
	IVIG use	11.70	2.26–60.54	< 0.001
	Steroid dose	1.03	1.00-1.05	0.005

ALT; alanine aminotransferase, AST; aspartate aminotransferase, DM; dermatomyositis, Hb; hemoglobin, ILD; interstitial lung disease, IVIG; intravenous immunoglobulin, MSA; myositis specific autoantibody, PLT; platelet count

### Risk factors for death due to dermatomyositis-associated spontaneous intramuscular hemorrhage

Our patient was discharged 2 weeks after the diagnosis of SIH. In 24 SIH patients, the hemorrhagic death rate was 50%. There was no statistical difference between those who died and not died due to hemorrhage in terms of clinical, laboratory and treatment. We also did not find an independent risk factor for bleeding-related death.

## Discussion

SIH is a rare complication in the course of DM. As far as we know that this is the first study in the literature to determine the frequency and risk factors for SIH development in DM patients. In our DM cohort, the SIH incidence rate was 0.238/100 patient years (95% CI 0.006–1.256). We have identified male sex, early stage of disease, the presence of dysphagia and ILD, anti-MDA5 positivity, anti-Ro52 positivity, heparin use, IVIG use and high steroid dose as risk factors for SIH development in our analysis.

There is not much information about the frequency of SIH. Case reports and a case series are defined in the literature. Xu et al., based on a literature review by presenting 7 MDA5 positive SIH patients, analyzed potential prognostic factors for SIH in advanced DM patients. They found the frequency of SIH in MDA5 + DM cohorts to be about 1% [5]. We have determined the frequency of SIH to be about 2% in our cohort.

It is very crucial for clinicians to know which stages of DM result in the development of SIH. In our DM cohort, our median follow-up time was 36 months. In our analysis, we found that SIH development was in the first month of DM. The development of muscular hemorrhage in the early period of the diagnosis of DM can be explained by the fact that the activity of DM is more intense in this period possibly due to microvascular changes.

When SIH develops, especially in the early stages of bleeding, the patient may be asymptomatic or complain about myalgia and swelling. Findings can range from asymptomatic anemia to hemorrhagic shock [4]. When DM patients describe regional severe muscle pain in the early stages of the disease, SIH must necessarily come to mind and an appropriate imaging tool should be performed to identify SIH. The hematoma may be localized to the limb, trunk, or abdomen. In our analysis, the most common hematoma site in 24 SIH patients was iliopsoas (41.7%). From a diagnostic point of view, the most commonly used diagnostic method in patients was CT (87.5%). Magnetic resonance imaging (MRI) and USG were also used [5–18]. When looking at the literature, angiography is used for diagnostic purposes, especially in patients with severe bleeding [5, 14, 16]. In patients with subscriber hemorrhage, angiography, both diagnostic and embolization-wise, may be a suitable option [5, 14].

The development of SIH seems to be multifactorial. We examined risk factors in terms of clinical and laboratory findings. Although DM appears to be more common in women, we have shown that male sex and early stages of disease are important clinical risk factors for SIH. We found the presence of dysphagia and ILD as other clinical risk factors for the development of SIH. Muscle enzymes that may indicate DM disease activity, increased CRP and the presence of MSAs and myositis-related antibodies are also tests that can provide information about the disease prognosis. Although Xu et al. noted that high CK values were associated with intramuscular hemorrhage, our analysis showed that CK levels at the time of diagnosis did not predict SIH development [5]. In addition, MDA5 antibody and anti-Ro52 positivity were important predictive factors in the development of SIH. The presence of MDA5 antibodies has a distinct significance for DM patients. The MDA5 antibody is not only diagnostic but also a leading antibody in terms of DM prognosis, disease activity [4, 21]. However, there is not enough information about the importance of MDA5 positivity in the pathogenesis of SIH. Xu et al. suggested that MDA5 positivity was associated with SIH [5]. This study only tried to determine prognostic factors in SIH patients with DM by analyzing patients who died and did not die. In this review, the mortality of patients with deep muscle hematoma was found to be significantly higher than that of patients with only superficial muscle hematoma [5]. We found that the presence of MDA5 antibody poses a risk of approximately 16 times for the development of SIH. MDA5 antibody was found to be associated with endothelial damage in DM patients [22]. Yang et al. also suggested that SIH development was a rare complication associated with the MDA5 antibody. It is also suggested that SIH is associated with peripheral inflammation of a single vessel, rather than extensive damage to capillary vessels around muscle tissues [4]. Another important antibody in terms of SIH is anti-Ro52. Xu et al. could not find any relationship between anti-Ro52 positivity and mortality, while our study found that the presence of anti-Ro52 may predict the development of SIH in patients with DM by 11.6 times but has not been predictive of death [5].

SIH has also been associated with some medications used to treat DM [5–7]. In practice, almost all DM patients need steroids. According to our study, the high dose of steroids rather than the use of steroids was a risk factor for the development of SIH. The steroid dose was much higher in SIH-developing patients in our cohort than in non-SIH patients. ILD was present in the majority of patients who developed SIH, which may have led to very high doses of steroids being administered to patients. In some cases, it is also seen that the dose of the steroid is increased after the bleeding is detected [8]. Could high-dose steroids really have caused bleeding or made it easier? While steroids disrupt vascular structural integrity and cause thinning and ecchymosis of the skin even at low doses, this side effect is exacerbated by higher doses [23].

In our analysis, half of the SIH patients received anticoagulants for various reasons. We found the use of heparin in univariate analysis as a risk factor for the development of SIH. But it was not a predictive factor for death. Our patient also had the use of heparin. Although heparin was continued while the hematoma was in progress (due to atrial fibrillation), or even later warfarin therapy under heparin (even when INR 3.4), bleeding did not recur and a new hematoma did not develop either. Despite our statistical identification of heparin use as a risk factor for SIH, heparin may have contributed to bleeding on the basis of possible microvascular damage caused by active DM.

Hanawa et al., suggested that intramuscular bleeding seen in DM may be due to increased vascular wall fragility, capillary vasculitis, steroid therapy, and prophylactic heparin use [6]. Why is hemorrhagic myositis seen especially in patients with DM? Besides the risk factors such as autoantibodies and drugs mentioned above, the main factor in the pathogenesis of SIH may be hidden in the pathogenesis of DM. In the case of developing SIH, the bleeding is only intramuscular. As well known, the DM pathogenesis differs from other IIMs. Atrophy of peripheral muscle fibers in DM is associated with microvascular capillary pathology. The most commonly identified vascular pathology in the muscle is a decrease and expansion in the number of some endomisal capillary vessels. In a study of DM muscle samples, endothelial damage was detected [24]. Evidence of immune-mediated microvascular damage may be the cause of bleeding in patients with DM. However, the event does not seem to be limited to the microvascular area. According to our analysis, some of the patients also had bleeding in medium vessels in angiography. SIH in the course of DM may also occur due to microaneurysms [19]. Kono et al. suggested that the presence of MDA5 antibodies could represent a possible risk of middle-vessel vasculitis in patients with DM [15]. However, it is still unclear how the medium-sized blood vessels in the perimycium are damaged in patients with DM [3].

When analyzing the risk variables, we believe that the development of SIH in DM is caused by multiple factors. Specifically, we can propose that microvascular damage occurred during the initial active phase of the disease as a result of the presence of certain antibodies, including anti-MDA5 and anti-Ro52. ILD’s existence has led to an excessive steroid dose, potentially causing bleeding due to increased tissue fragility from steroids. Heparin may also increase the likelihood of bleeding in the presence of damaged tissue.

Although SIH in the course of DM is rare, its mortality is very high. Although we found some risk factors for the development of SIH, we did not find predictive factors for mortality. There is no established recommendation on SIH treatment. However, stopping anticoagulants and appropriate blood product transfusions should be applied. According to the condition of the bleeding, angiographic vascular embolization should be evaluated in the patient’s particular. Although we have established a relationship between steroid dosage and SIH development, it should be noted that the main treatment of DM is steroid, the patient’s steroid dose should be individualized in the presence of a vital DM organ involvement.

Although our study was the first to identify risk factors for SIH in patients with DM, it had some limitations. First, it was a retrospective study. The second was that the number of patients was relatively small. Another was that MSAs were not studied in all patients. Another limitation was using data from SIH patients in the literature. The last was that the treatments applied to patients were heterogeneous. This may have affected the results.

As a result, in the course of DM, SIH is a rare complication with a very high mortality rate. Rheumatologists should be cautious about the development of SIH in DM patients with lung involvement, presence of dysphagia, heparin used, very high doses of steroids given, positive for MDA5 antibody, and/or anti-Ro52 especially in the early stage of the diagnosis of DM. It should be remembered that patients can be asymptomatic. Larger-scale prospective studies are needed in the future to more clearly demonstrate the effects of MSAs (especially anti-MDA5), anti-Ro52, drugs, and other disease-related features in the development of SIH.

## Data Availability

All data underlying the results are available as part of the article and no additional source data are required. Data are available upon reasonable request to the corresponding author.

## Declarations

### Previous related congress abstract publication

The authors declare that this article or its part has not been copied or published as a congress abstract publication before.
